# Role of cold atmospheric plasma alone or combined with conventional surface treatments on shear bond strength of 3Y-TZP and 5YSZ ceramics bonded with composite resin

**DOI:** 10.2340/biid.v13.45567

**Published:** 2026-03-30

**Authors:** Ahmad Abdulkareem Alnazzawi, Mohamed F Aldamaty, Mohammed H. AbdElaziz, Ahmed Yaseen Alqutaibi, Ahmed E. Farghal, Jamal Qernas Almarashi, Abdel-Aleam H. Mohamed, Muhammad Sohail Zafar

**Affiliations:** a Department of Substitutive Dental Science, College of Dentistry, Taibah University, Al-Madinah Al-Munawwarah, Saudi Arabia; b Department of Fixed Prosthodontics, Faculty of Dental Medicine, Al-Azhar University, Cairo, Egypt; c Department of Restorative and Aesthetic Dentistry, College of Dentistry, Almaaqal University, Basrah, Iraq; d Physics Department, College of Science, Taibah University, Al-Madinah, Saudi Arabia; e Department of Clinical Sciences, College of Dentistry, Ajman University, Ajman, United Arab Emirates; f Center of Medical and Bio-allied Health Sciences Research, Ajman University, Ajman, United Arab Emirates; g School of Dentistry, University of Jordan, Amman, Jordan

**Keywords:** adhesive protocol, dental ceramic, surface treatment, translucent zirconia, zirconia primer

## Abstract

**Objective:**

To evaluate the role of cold atmospheric plasma (CAP), alone or in combination with conventional surface treatments, on the shear bond strength (SBS) and failure mode of 3Y-TZP and 5YSZ ceramics bonded to composite resin using two adhesive protocols.

**Materials and methods:**

A total of 200 zirconia specimens (3Y-TZP and 5YSZ) were assigned to five surface-treatment groups: No treatment (control), airborne-particle abrasion (APA), CAP, APA + CAP, and hydrofluoric acid (HF) + CAP. Each group was further divided according to the use of a primer. The zirconia specimens were bonded to composite resin using a self-adhesive cement. After artificial aging, SBS testing and failure-mode analysis were performed. Data were analyzed using appropriate parametric tests (α = 0.05).

**Results:**

Compared with the control group, surface treatments significantly improved SBS (*p* < 0.001). Applying self-adhesive cement with surface primer further improved SBS (*p* < 0.001). Different yttria contents of zirconia materials did not impact SBS (*p* > 0.05). There was no benefit to combining APA and CAP or CAP and HF. Failure modes were predominantly adhesive in the control group and mixed on treated zirconia surfaces.

**Conclusions:**

CAP provides bond strength comparable to APA while potentially minimizing surface damage, supporting its use as a conservative surface-treatment option. Additional combined or aggressive surface treatments do not offer further clinical benefit for bonding zirconia restorations.

## Introduction

Ceramics are the most desirable and applicable materials in restorative and prosthetic dentistry, particularly zirconia [1]. Since the introduction of zirconia at the beginning of the third millennium, it has undergone several developmental stages at both chemical and structural levels [2]. The first two generations of zirconia contain approximately 3 mol% yttria and are referred to as 3Y-TZP (3 mol% yttria-stabilized tetragonal zirconia polycrystal) [2]. This material has been utilized in a wide range of dental applications, from simple restorations to complete arch dental prostheses, due to its high biocompatibility, relatively good esthetic value, and high flexural strength. Additionally, the transformation toughening inhibits crack propagation, thereby increasing fracture strength [3]. Nevertheless, 3Y-TZP is relatively opaque compared to other ceramics. To address this limitation, a third generation of zirconia was introduced, characterized by increased yttria contents such as 4 and 5 mol% Yttria-stabilized zirconia (YSZ) [2]. Translucent zirconia is more suitable for esthetic restorations, particularly 5YSZ, which has been employed for anterior esthetic veneers and full crowns [4]. This increase in yttria content results in a higher cubic crystalline phase, which is responsible for the enhanced translucency in 4YSZ and 5YSZ [5, 6].

The reliable and durable bonding of dental restorations and prostheses necessitates high-adhesion potential surfaces. Some restorative materials, such as resinous restorations, require no surface treatment before adhesive procedures, whereas other restorations, such as glass and oxide ceramics, require surface preparation before adhesion. The most effective treatment for glass ceramic materials is hydrofluoric acid (HF) etching [7]. However, oxide ceramics, particularly zirconia, characterized by having an inert structure that is resistant to chemical etching, and airborne particle abrasion (APA) is deemed as the most effective and recommended surface treatment [8]. Despite its widespread clinical application, this method can adversely affect the zirconia surface. Some particles might remain embedded within the zirconia surface after abrasion, leading to the generation of multiple microcracks and premature transformation toughening, possibly weakening the surface strength of the treated specimens [9, 10].

Several laboratory investigations assessed the viability of efficient and more inert surface treatment methods, including cold atmospheric plasma (CAP) [11–13] high-temperature HF acid solution etching [14–16], and the application of phosphate monomer-containing primers [17–19]. A notable example of zirconia primers is Z-prime, which contains the 10-methacryloyloxydecyl dihydrogen phosphate (MDP) phosphate monomer and the biphenyl di-methacrylate (BPDM) carboxyl monomer, believed to exert a synergistic effect that results in elevated bond strength [20]. In recent years, plasma technology has emerged as a method to improve the performance of biomaterials [11–13]. Plasma, identified as the fourth state of matter, comprises atoms, molecules, and highly energized radicals. This surface treatment includes both thermal and nonthermal (cold) variants, with the cold plasma type operating at gas temperatures close to room temperature under atmospheric pressure [21]. Atmospheric plasma is employed for surface activation, influencing the uppermost atomic layers of the material’s surface. An untreated surface typically exhibits minimal polar groups capable of interacting with liquids, whereas plasma treatment introduces such polar end groups onto the surface molecules [21, 22]. The utilization of CAP may present a promising methodology, as this treatment enhances the wettability of ceramic surfaces, potentially enhancing bonding potential [23, 24].

The present study aimed to evaluate the role of CAP, alone or in combination with conventional surface treatments, on the shear bond strength (SBS) and failure mode of 3Y-TZP and 5YSZ ceramics bonded with composite resin using two adhesive protocols. The null hypotheses were: (1) Various surface treatments would show comparable SBS values of 3Y-TZP and 5YSZ. (2) The type of monolithic zirconia would not significantly influence SBS. (3) Various luting protocols, with or without zirconia primers, would not impact on the SBS values.

## Materials and methods

### Study design and sample size calculation

Two hundred square-shaped zirconia specimens (100 for both materials; 13.3×13.3×3.3 mm^3^) were fabricated using 3Y-TZP (ceraMotion® Z HT Multishade, Dentaurum, Germany) and 5YSZ (ceraMotion® Z Cubic Multishade, Dentaurum, Germany). The sample size was calculated to assess the impact of five different surface treatments on the SBS of two zirconia tested types (3Y-TZP and 5YSZ) when bonded to composite. The following test groups were assigned for experiment (1) Control (without any treatment), (2) airborne-particle abrasion (APA), (3) CAP, (4) APA and CAP, and (5) HF acid, 9.5% (HF) etching, and CAP, each with and without primer, resulted in a total of 10 experimental groups. Based on Xie et al. and according to G*Power statistical software (v. 3.1.9.4), more than 48 specimens may suffice to identify the large effect size (*f* = 0.51) with an actual power (1-β error) of 0.8 (80%) and a significance level (α error) 0.05 (5%) for a two-sided hypothesis test [25]. To increase study power, 200 specimens (100 for each material) were divided into surface treatment groups (*n* = 20) and primer subgroups (*n* = 10).

### Preparation of zirconia specimens

A precision cutting saw (IsoMet4000 microsaw, Buehler, Illinois, USA) was used to cut zirconia blanks into square-shaped specimens along with water coolant. A diamond disc (0.6 mm thick) was operated at 2500 rpm (feed rate of 10 mm/min). The dimensions of the cut specimens were carefully coordinated using a digital micrometer and a traveling stage. A 600-grit SiC mounted on an Automet 500 (Buehler, Esslingen, Germany) was used to wet-grind discs for 60 seconds. Prior to subsequent testing, prepared specimens were ultrasonicated and completely dried [26]. Zirconia specimens were placed in an infire HTC speed furnace (Sirona, Germany) for sintering following the manufacturer’s recommendations. After sintering, shrinkage of zirconia occurs by a ratio of 25%. Therefore, the dimensions of each sintered specimen were approximately 10×10×2.5 mm.

### Composite specimen fabrication

Custom-designed Teflon molds were used to standardize the dimensions and alignment of the composite resin specimens (*n* = 200). Mold (Z) comprised an inner square Teflon mold featuring a central aperture measuring 10 × 10 × 2.5 mm, specifically designed to accommodate the zirconia specimens. Mold (C) consisted of a circular, split Teflon mold having an internal square cavity possessing a surface area (6 × 4 mm), intended to hold the composite resin material directly above Mold (Z). An external ring was fabricated to stabilize the entire mold assembly.

A transparent glass slab was positioned under the mold, and a nanohybrid composite (Tetric N-Ceram, Ivoclar Vivadent AG, Liechtenstein) was incrementally applied within Mold (C). Each layer was densely compacted and light cured for 40s utilizing a halogen curing light (Elipar, 3M ESPE, Leicestershire, England) with the output power set at 1350 mW/cm². The maximum thickness for each increment was established at 2 mm. Upon removal of the molds and glass slab, an additional curing period of 20 seconds was implemented to ensure complete polymerization. Excess composite at the margins was trimmed using a micro-motor, and the samples underwent visual inspection for porosities and defects and were measured for standardization.

### Surface treatment of zirconia discs

**Figure 1:**
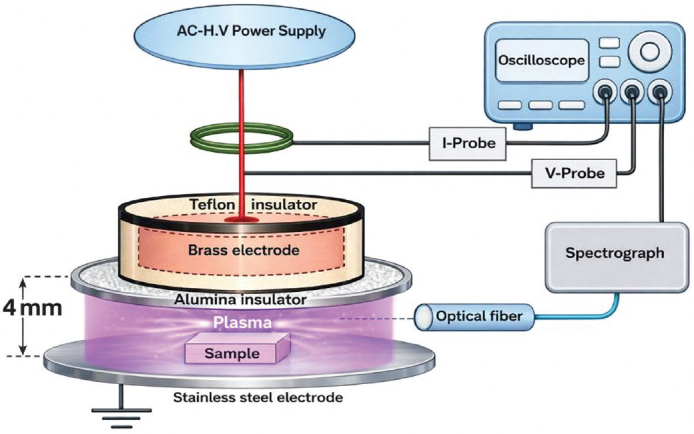
Diagram showing cold atmospheric plasma surface treatment.

Zirconia specimens were categorized into five groups (*n* = 20) based on the surface treatments:

1. **C** (control group); No additional surface treatment was applied.
2. **APA** (airborne particle abrasion): The zirconia surfaces were subjected to airborne particle abrasion for 20 s with 50 µm aluminum oxide (Al₂O₃) particles (Korox 50, BEGO, Bremen, Germany), maintaining 2 bar pressure from 10 mm [24]. A tailor-designed frame was employed to regulate the perpendicular orientation and distance between the zirconia specimens and the microblaster nozzle. The specimens were then ultrasonically cleaned and completely dried.
3. **CAP**: Zirconia specimens received CAP treatment before the application of the adhesive cement. Dielectric barrier discharge plasma system was utilized at atmospheric pressure [27]. This system comprises two metallic electrodes, spaced 4 mm apart. The upper electrode is constructed from brass (45 mm diameter) and covered in Teflon for safety, while the lower electrode is made of stainless steel (50 mm diameter, 3 mm thick) and is grounded. The upper electrode was connected to an alternating current power supply (Plasma Driver PVM500, Information Unlimited Co.), which delivers a high voltage (20 kV) and a frequency of 20 kHz in a sinusoidal signal (33.33 kΩ resistor) to limit current [27]. Each specimen was placed on the grounded lower electrode and treated with CAP for 5 minutes (Figure 1).
4. **APA + CAP** (combination of APA and CAP): Zirconia specimens were first subjected to APA as in group 2, followed by CAP treatment as outlined in group 3.
5. **HF + CAP:** (Combination of HF acid etching and CAP), Specimens were subjected to etching for 1 hour at room temperature using HF acid gel (9.5%) (Porcelain Etch Gel, Bisco, USA) [15], then washed, completely dried, and followed by CAP treatment as described in group CAP.

### Scanning electron microscopy

An environmental scanning electron microscope (Thermo Fisher Scientific™ Inc., USA) was used to analyze randomly selected specimens (*n* = 2) from each surface treatment group (30 kV accelerating voltage, 2500x magnification). Randomization was performed by assigning each specimen a numerical code, followed by selection using a computer-generated random number list. The complimentary software (Thermo Scientific™ Maps™ Software) was used to adjust the image contrast and analysis.

### Adhesive cementation procedure

A zirconia primer (Z-Prime Plus; BISCO, Schaumburg, Illinois, USA) was coated for 10 s (control and four experimental groups, total *n* = 100), followed by a drying period of 15 seconds. Zirconia and composite discs were bonded utilizing a universal luting resin with a cleanup indicator (Maxcem Elite Chroma, Kerr Corporation, Italy) under a 0.2-kg constant load to ensure uniform cementation [28, 29]. Excess luting was carefully wiped using a cotton pellet, followed by light curing each side of margins for 40s using a halogen curing light (Elipar, 3M ESPE, Leicestershire, England) at an intensity of 1350 mW/cm². For the remaining specimens from control and four experimental groups (*n* = 100), identical procedural steps were applied, however, without applying the zirconia primer. Following the adhesive cementation procedure, all samples were placed in distilled water 24 h at 37°C prior to thermocycling.

### Thermocycling of specimens

All samples underwent a cumulative total of 10,000 cycles in a thermocycler (SD Mechatronic, Westerham, Germany), oscillating between 5°C and 55°C. Each temperature was maintained for a dwell time (30s), with a transfer time of 10 s between temperature changes [26].

### SBS testing

A universal mechanical tester (Instron, England) with a mono-beveled chisel attachment was utilized for assessing the assembly of the zirconia/composite specimens. The testing protocol involved the application of a load (5000 g) with a constant crosshead speed (0.5 mm/min). The SBS (τ) was evaluated using the following equation:

τ = F/A

where τ denotes SBS in MPa; F and A represent the force and the bonding area (mm²), respectively. The bonded area is obtained by the area of the circle (A= πr^2^ = 28.27 mm^2^). The resulting findings were recorded using BlueHill software (Universal Instron, England).

### Analysis of failure mode

The modes of failure were systematically categorized utilizing an optic microscope (Nikon SMZ745T, Japan) at a magnification of 30×. Failures were classified into three distinct categories: adhesive failures, which occur at the resin cement/zirconia interface; cohesive failures, which take place within either the composite or the zirconia; and mixed failures, which involve a combination of both adhesive and cohesive mechanisms.

### Statistical analysis

Data were statistically analyzed using SPSS (v.20, SPSS Inc., Chicago, IL, USA). Numerical findings were expressed as means ± standard deviations, accompanied by confidence intervals and ranges. The normality of the data distribution was assessed using the Kolmogorov-Smirnov and Shapiro-Wilk tests. Since the data conformed to a normal distribution, group comparisons were performed employing a one-way Analysis of Variance (ANOVA) and Bonferroni’s post-hoc test. Intragroup comparisons were performed using the independent *t*-test, while a multi-way ANOVA was executed to examine interactions among study variables. The *p*-values were analyzed as two tailed, with statistical significance established at *p* ≤ 0.05.

## Results

### Scanning electron microscopy

**Figure 2:**
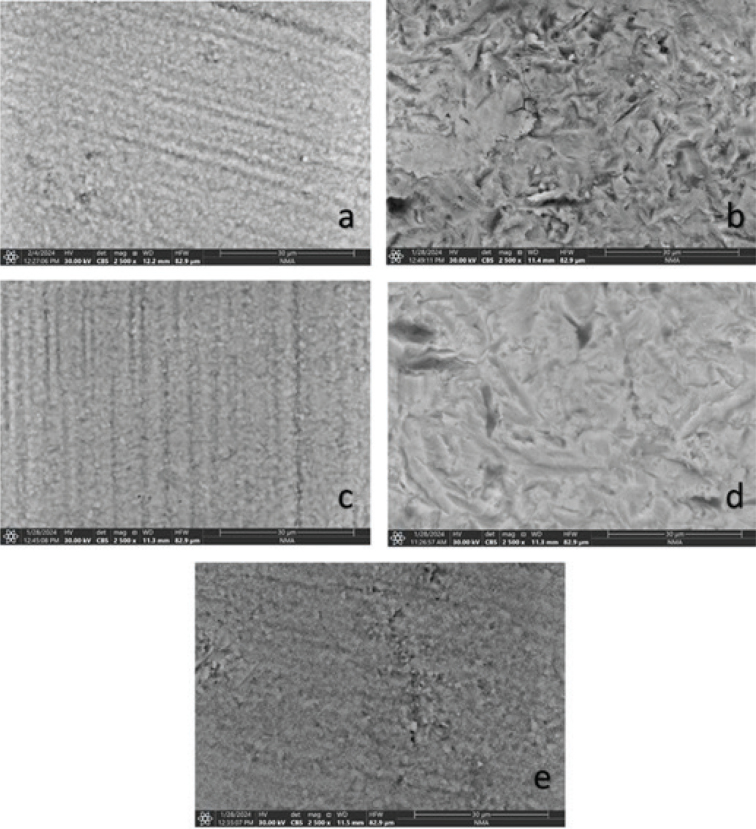
Representative SEM images: (a) Control group specimen without surface treatment. (b) Air particle abrasion surface treatment. (c) Cold atmospheric plasma specimen. (d) Air particle abrasion followed by cold atmospheric plasma. (e) Hydrofluoric acid etching followed by cold atmospheric plasma.

Different surface treatments revealed different surface topography under environmental scanning electron microscopy. The control untreated group showed a surface without markable surface roughness, the surface characterized by parallel grooves that could be representative of the sandpaper polishing of the specimens, Figure 2a. The specimens treated with APA revealed high surface topographic change; the surface roughness is more prominent, and there are also noticeable ditches representative of sharp edges of Al_2_O_3_ particles impingement on the surface, Figure 2b. CAP specimen showed no remarkable changes in the surface topography, Figure 2c. Subjecting the specimens to APA followed by CAP did not significantly change the appearance compared to solely APA treatment; however, the surface appears less random and smoother while preserving the APA-generated irregularities (Figure 2d). HF acid application before CAP did not produce a significant alteration of the surface; it remained comparable to the control specimens although the zirconia granules appeared more pronounced than in the other specimens (Figure 2e).

### SBS

All surface treatments demonstrated a significant increase in SBS compared with the control group (*p* < 0.001). Similarly, self-adhesive cement applied in conjunction with a surface primer significantly enhanced SBS compared to groups that did not use a surface primer (*p* < 0.001). In the case of the 3Y-TZP groups, all specimens subjected to surface treatment exhibited comparable mean SBS values within the no primer groups (*p* > 0.05). However, within the primer groups, the APA and CAP surface treatments demonstrated statistically significantly higher SBS values than the APA + CAP and CAP + HF treatments (*p* < 0.001). For the primer 3Y-TZP groups, the mean SBS values ranged from 18.34 ± 1.68 MPa to 21.90 ± 3.07 MPa, with the latter representing the highest SBS value observed among all groups. Conversely, for the no primer 3Y-TZP groups, the SBS values ranged from 14.01 ± 1.37 MPa to 14.79 ± 1.42 MPa. The lowest SBS values for the 3Y-TZP specimens were associated with control specimens lacking primer, recorded at 4.72 ± 0.96 MPa (*p* < 0.001).

In the 5YSZ groups, all specimens with surface treatment exhibited comparable mean SBS values within the primer (*p* > 0.05) and no primer groups (*p* > 0.05). For the primer 5YSZ groups, the mean SBS values ranged from 18.03 ± 2.18 MPa to 19.36 ± 1.48 MPa. In contrast, for the no primer 5YSZ groups, the SBS values ranged from 13.54 ± 0.91 MPa to 14.79 ± 1.42 MPa. The lowest SBS values for the 5YSZ specimens were associated with control specimens without primer, recorded at 4.62 ± 1.04 MPa (*p* < 0.001). Descriptive statistics, along with comparisons among various groups and their interactions, are presented in Tables 1 and 2. The statistical power of the study, based on different variables, ranged from 0.94 (94%) to 1 (100%).

**Table 1:** Effect of various surface treatments and bonding protocols on SBS (data presented as mean and standard deviation; MPa).

Material	Surface treatment	Bonding protocol			
		No primer		Primer	
		Mean	Std. dev	Mean	Std. dev
**3Y-TZP**	C	4.72 ^E^	0.96	6.78 ^D^	1.04
	APA	14.20^C^	2.21	21.90^A^	3.07
	CAP	14.79^C^	1.42	20.31 ^A^	1.76
	APA + CAP	14.60 ^C^	1.36	19.36 ^B^	1.90
	HF + CAP	14.01 ^C^	1.37	18.34 ^B^	1.68
**5YSZ**	C	4.62 ^E^	1.04	6.86 ^D^	0.84
	APA	14.26 ^C^	1.59	18.03 ^B^	2.18
	CAP	13.23 ^C^	1.24	19.36^B^	1.48
	APA + CAP	13.54 ^C^	0.91	18.95 ^B^	1.52
	HF + CAP	13.85 ^C^	1.86	18.16 ^B^	1.12

Uppercase superscript letters indicate the significant differences between all groups, the letter A represents statistically significantly the highest SBS values, while the letter E indicates statistically significantly the lowest SBS values among all groups.

APA: Air particle abrasion, C: Control, CAP: Cold atmospheric plasma, APA + CAP: Cold atmospheric plasma after airborne particle abrasion, HF + CAP: Cold atmospheric plasma with hydrofluoric acid.

**Table 2:** Interaction of variables analyzed using the multiple-way ANOVA.

Source	Type III sum of squares	df	Mean square	F	P value	Partial eta squared	Observed power
**Surface ttt**	3852.05	4.00	963.01	368.59	0.0001*	0.891	1.00
**Primer**	1068.10	1.00	1068.10	408.82	0.0001*	0.694	1.00
**Zr (3Y & 5Y)**	33.09	1.00	33.09	12.66	0.0001*	0.066	0.94
**Surface ttt × Primer**	91.17	4.00	22.79	8.72	0.0001*	0.162	1.00
**Surface ttt × Zr**	24.61	4.00	6.15	2.35	0.056 ns	0.050	0.67
**Primer × Zr**	3.15	1.00	3.15	1.20	0.274 ns	0.007	0.19
**Surface ttt × Primer × Zr**	37.57	4.00	9.39	3.60	0.008*	0.074	0.87

3Y: 3 mol% yttria-partially stabilized monolithic zirconia. 5Y: 5 mol% yttria-partially stabilized monolithic zirconia. Significance level *p* ≤ 0.05,

* significant. ns: nonsignificant.

### Mode of failure

**Figure 3:**
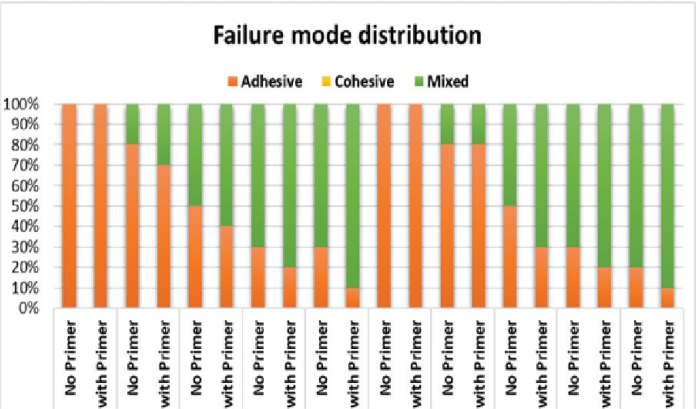
Comparison of the distribution pattern of the mode of failure of 3Y-TZP and 5YSZ.

**Figure 4:**
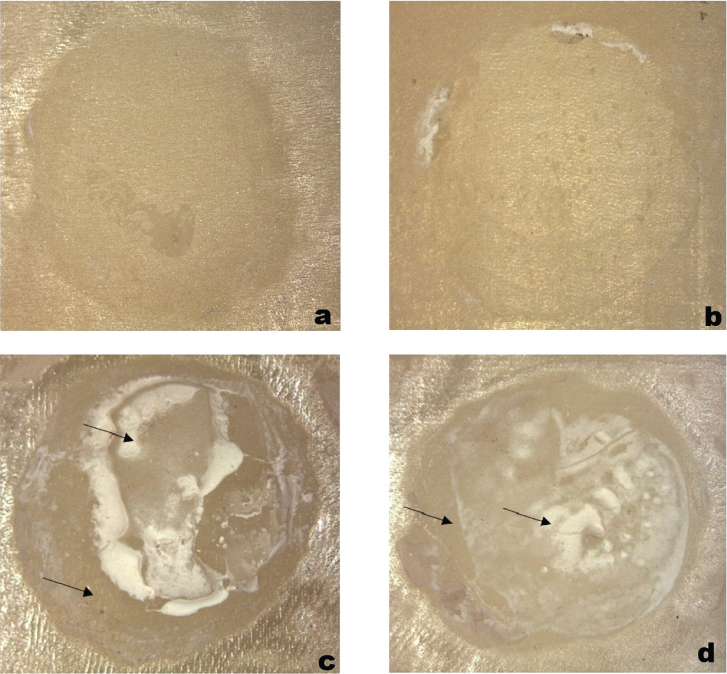
(a and b) Complete adhesive failure at the zirconia surface. (c) Mixed failure showing adhesive failure at a composite surface, cohesive within the cement surface, and adhesive failure at the zirconia surface. (d) Mixed failure mode showing adhesive failure at the composite resin cement interface and cohesive failure within the composite material.

In the present study, the identified failure modes were exclusively adhesive or mixed failures, without instances of pure cohesive failures. Furthermore, within the mixed failure modes, the cohesive failure components were situated within the composite material. Notably, neither pure nor mixed cohesive failure modes were associated with zirconia materials. The control groups demonstrated only adhesive failures, suggesting an early detachment of the cement layer from the untreated zirconia surfaces. Conversely, all groups with surface treatment exhibited both adhesive and mixed failures (Figures 3 and 4).

## Discussion

This study evaluated the influence of surface treatments and luting protocols on SBS of two monolithic zirconia materials (3Y-TZP and 5YSZ) bonded to composite resin with a self-adhesive resin cement. Surface treatment significantly enhanced the SBS of zirconia, leading to the rejection of the first null hypothesis. In contrast, the yttria content of zirconia did not significantly influence SBS, and therefore the second null hypothesis was accepted. The application of a zirconia primer significantly improved SBS, resulting in rejection of the third null hypothesis.

Achieving reliable and durable adhesion to zirconia remains a clinical challenge due to its chemically inert surface and absence of a glassy phase. APA remains the most commonly used surface treatment for zirconia, as it increases surface roughness and surface area, thereby enhancing micromechanical interlocking and surface wettability [30, 31]. In the present study, APA using 50 µm alumina particles at 0.2 MPa effectively improved SBS, consistent with previous reports [24, 32].

Beyond micromechanical retention, CAP modifies zirconia surfaces primarily through physicochemical mechanisms. CAP increases surface energy by removing organic contaminants and introducing highly reactive oxygen- and nitrogen-containing functional groups, such as hydroxyl and peroxide radicals [31, 33, 34]. These plasma-induced radicals significantly enhance surface wettability and promote improved spreading and penetration of primers and resin cements. Unlike APA, CAP alters surface chemistry without inducing additional surface damage, which may reduce the risk of microcrack formation in zirconia [35].

Although CAP combined with APA did not result in statistically significant SBS improvements compared to CAP alone, the observed trend suggests a complementary effect. These findings are consistent with those of Inokoshi et al. [36], who reported that CAP primarily enhances chemical bonding rather than surface roughness. Therefore, the principal advantage of CAP lies in optimizing the chemical reactivity of zirconia surfaces rather than further increasing topographical irregularities.

HF etching was found to be ineffective for zirconia surface treatment, confirming previous evidence that zirconia lacks an etchable glass phase and is resistant to acid dissolution [37, 38]. Even prolonged exposure to highly concentrated HF produces minimal surface alteration without meaningful improvement in bond strength. Accordingly, the present findings further support the clinical consensus that HF etching is incompatible with zirconia restorations.

Chemical bonding to zirconia is predominantly achieved through phosphate monomer-containing primers, particularly 10-MDP. The 10-MDP molecule forms stable chemical bonds with zirconium oxide via its phosphate group while copolymerizing with resin cement through its methacrylate group [39–41]. In the current study, the application of a 10-MDP–containing primer significantly improved SBS, even after artificial aging. Although the manufacturer suggests potential synergistic effects of multiple phosphate monomers, recent evidence indicates that bond durability is primarily attributed to 10-MDP alone [39].

The absence of significant SBS differences between 3Y-TZP and 5YSZ observed in this study aligns with some previous reports although conflicting findings exist in the literature [42]. Variations among studies may be attributed to differences in aging protocols, luting agents and testing methods [43]. The modest SBS values observed in untreated control groups further emphasize that surface treatment and primer application are essential for zirconia restorations that rely on adhesive retention [34, 44].

Failure mode analysis supported these findings, as untreated zirconia predominantly exhibited adhesive failures, whereas treated groups showed mixed failure patterns indicative of improved interfacial bonding [45–48]. CAP-treated surfaces likely enhance primer–zirconia interaction by increasing surface polarity and chemical reactivity, thereby improving adhesion quality [47].

From a clinical perspective, CAP offers promising adjunctive surface treatment for zirconia restorations in both dental laboratories and clinical settings. CAP devices operate at room temperature, require short application times, and do not alter zirconia surface morphology, making them suitable for chairside or laboratory use. When combined with 10-MDP primers, CAP may improve bonding reliability while minimizing mechanical damage associated with aggressive air abrasion. However, practical considerations such as equipment cost, training requirements, and workflow integration must be addressed before widespread clinical adoption.

This study is limited by the exclusive use of a single self-adhesive resin cement, which may behave differently from adhesive luting systems. Additionally, only two zirconia compositions were evaluated; inclusion of 4YSZ could provide a broader comparison. Finally, as an in vitro investigation, the findings may not fully replicate intraoral conditions, and long-term clinical studies are needed to validate these results.

## Conclusions

The present in vitro study concluded that airborne particle abrasion utilizing alumina in conjunction with the application of a primer resulted in the highest SBS of zirconia to resin composite. CAP provides bond strength comparable to APA while potentially minimizing surface damage, supporting its use as a conservative surface-treatment option. Additional combined or aggressive surface treatments do not offer further clinical benefit for bonding zirconia restorations. The bonding efficacy was significantly enhanced when surface treatments were utilized in conjunction with primers containing MDP. This combination indicates the establishment of a reliable bond even after exposure to artificial aging conditions. The present study identified no significant difference in monolithic zirconia with varying yttria content, confirming that bond strength was primarily influenced by the surface treatment and adhesive protocol used, rather than the yttria content or the mechanical-physical properties of zirconia ceramic materials.

## Declarations

## Conflicts of interest/Competing interests

The authors declare that they have no conflicts of interest.

## Availability of data and material

The data will be made available as per requirement.

## Code availability

Not applicable.

## Ethics approval

Not applicable.

## Consent to participate

Not applicable.

## Consent for publication

Not applicable.
